# Supplementary material for: Formation of Abasic Oligomers in Nonenzymatic Polymerization of Canonical Nucleotides
Source: Life (Basel). 2019 Jul 4;9(3):57. doi: 10.3390/life9030057 (PMC6789551; doi:10.3390/life9030057)

APPENDIX

Following mass spectra were acquired at ELSI, Japan in collaboration with Dr. Hongo. Samples were analyzed as described in Section 3.1 and 3.2.

A.1 : Mass spectrum of AMP oligomerization reaction

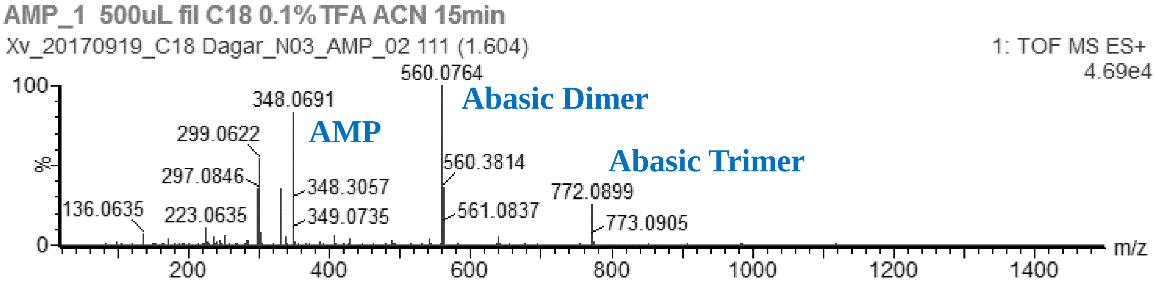

A.2 : Mass spectrum of GMP oligomerization reaction

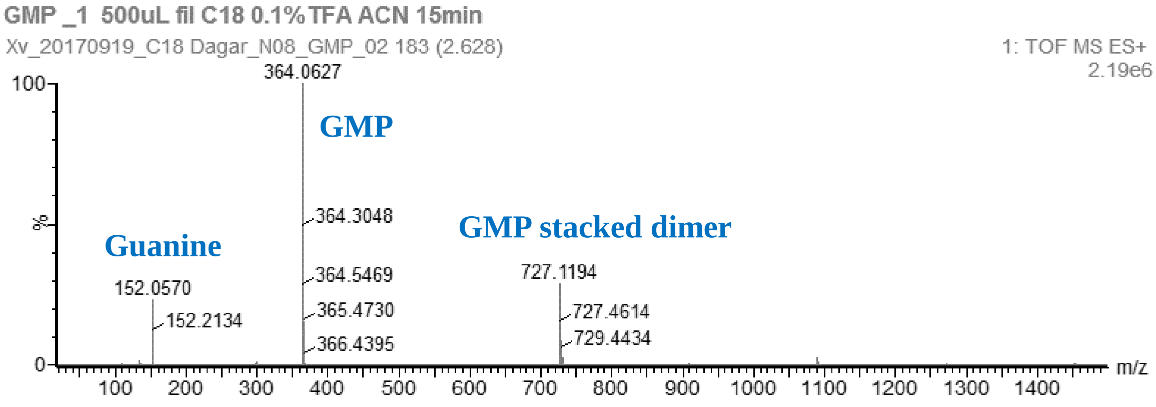

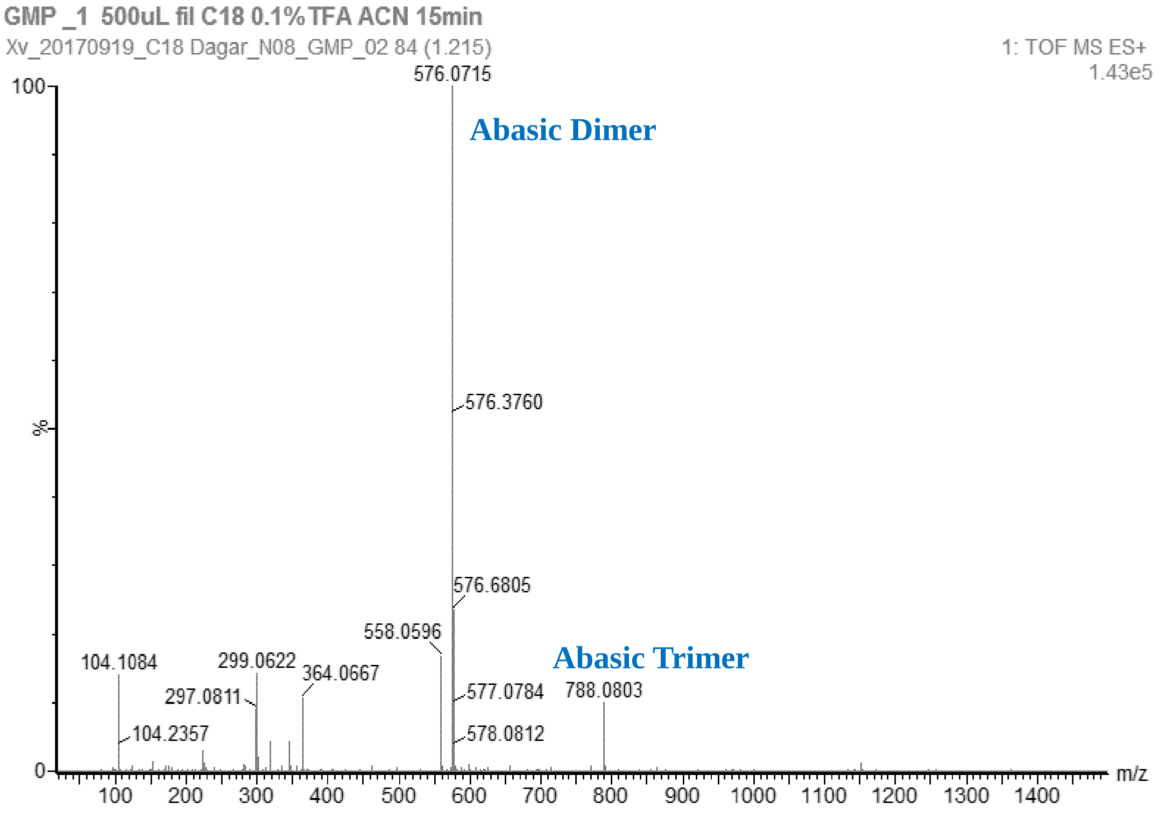

A.3 Mass spectrum of UMP oligomerization reaction

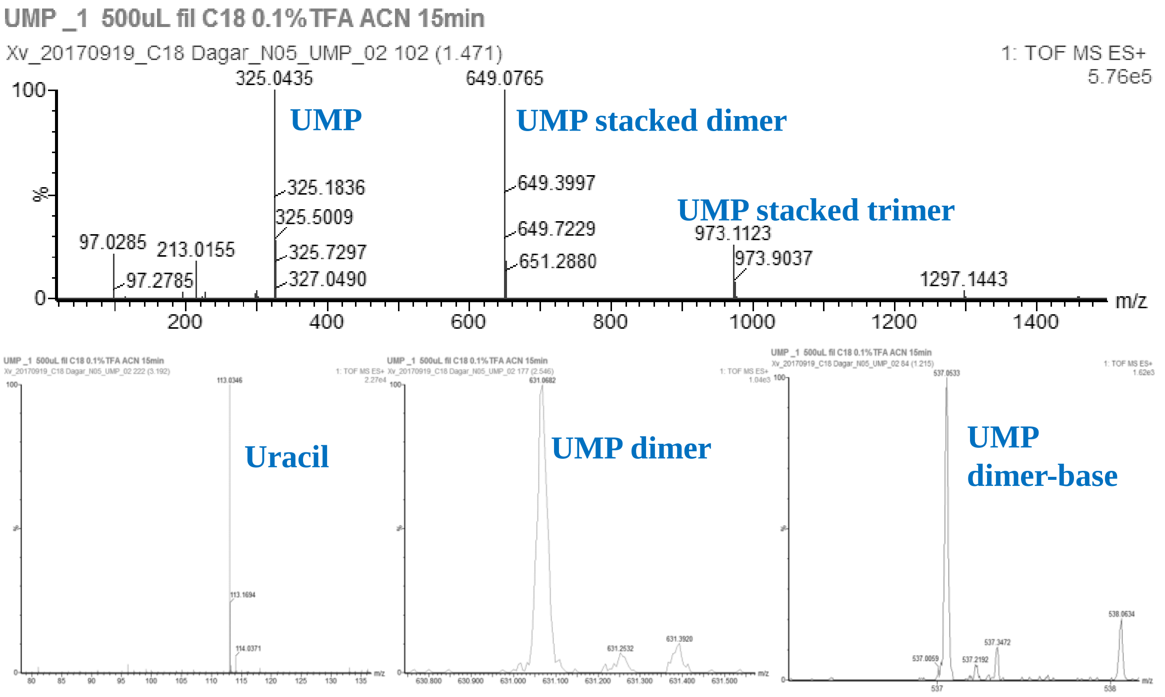

A.4 Mass spectrum of CMP oligomerization reactions

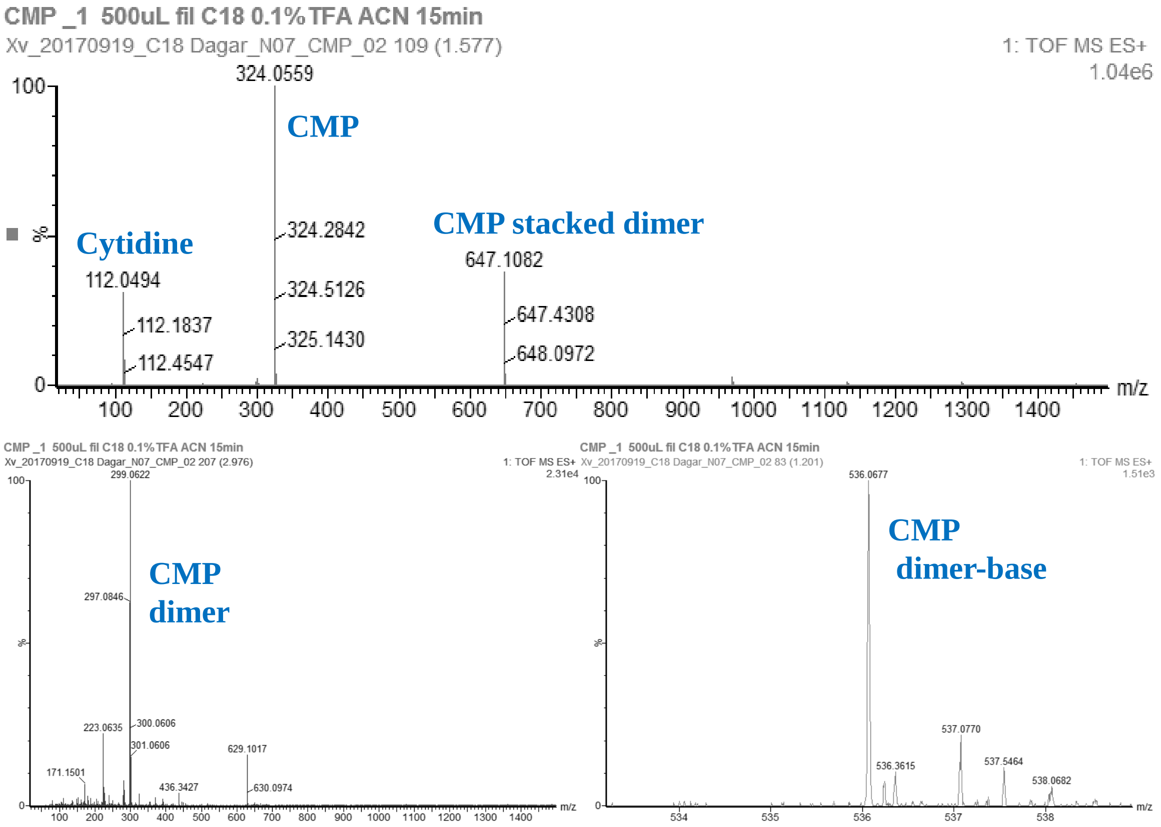

A.5 : Mass spectrum of AMP+UMP reactions

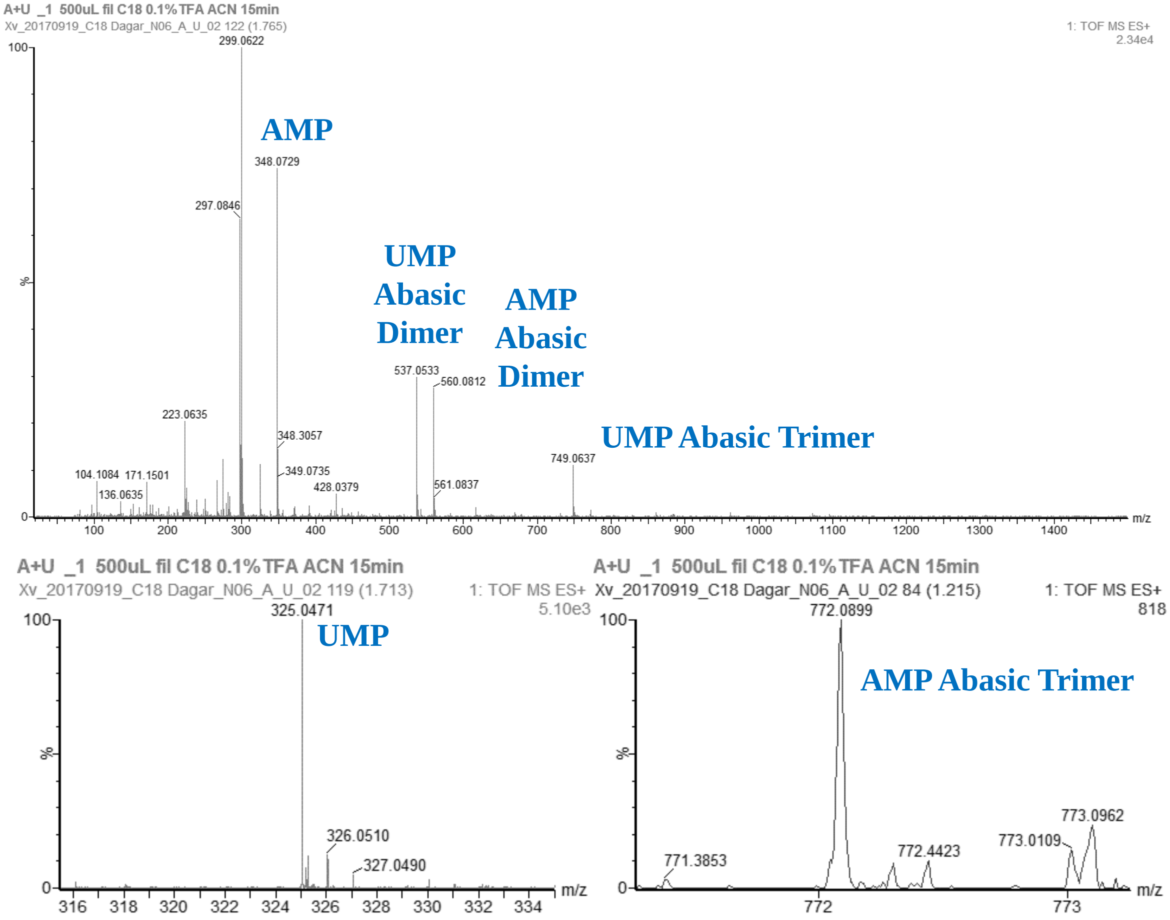

A.10: Mass spectrum of GMP+CMP reactions

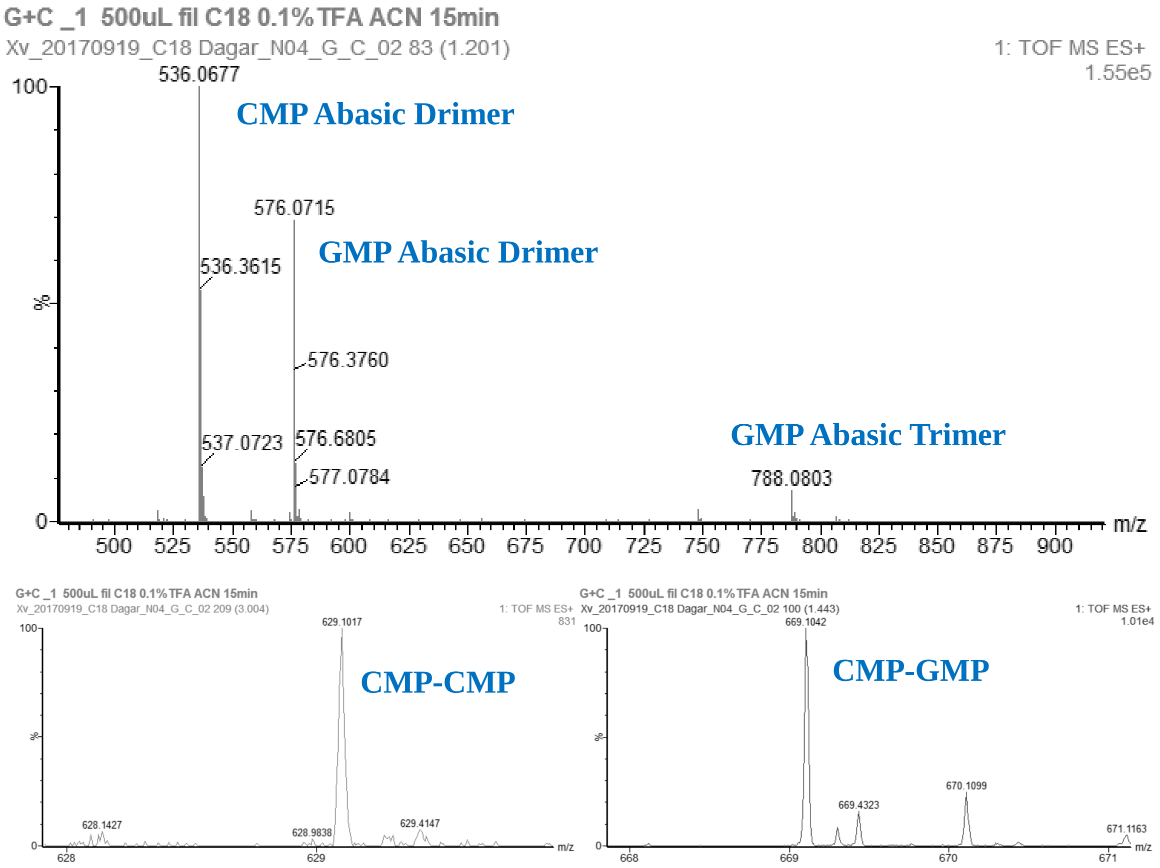

Supplement: Supplementary file 1 [file life-09-00057-s001.zip › life-528552-Supplementary Materials File 1.pdf]
