# Supplementary material for: Formation of Abasic Oligomers in Nonenzymatic Polymerization of Canonical Nucleotides
Source: Life (Basel). 2019 Jul 4;9(3):57. doi: 10.3390/life9030057 (PMC6789551; doi:10.3390/life9030057)

Supplementary Figure S1: HPLC chromatograms of oligomerization reactions. (Reproduced from [4])

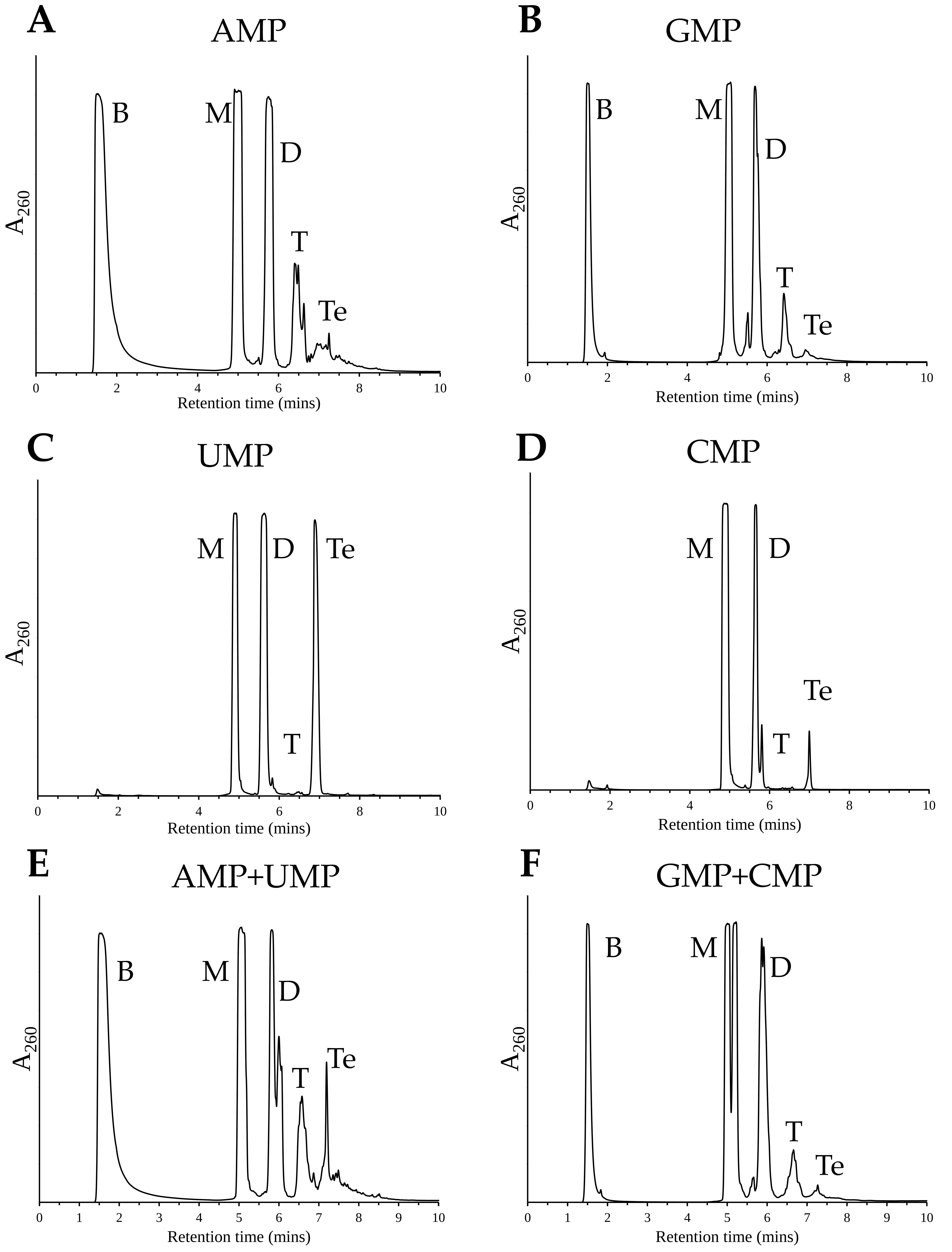

Legend: B: Breakdown; M: Monomer; D: Dimer; T: Trimer; Te: Tetramer

Supplementary Figure S2: Mass spectrums of nucleotide monomer controls showing presence of free bases due to fragmentation.

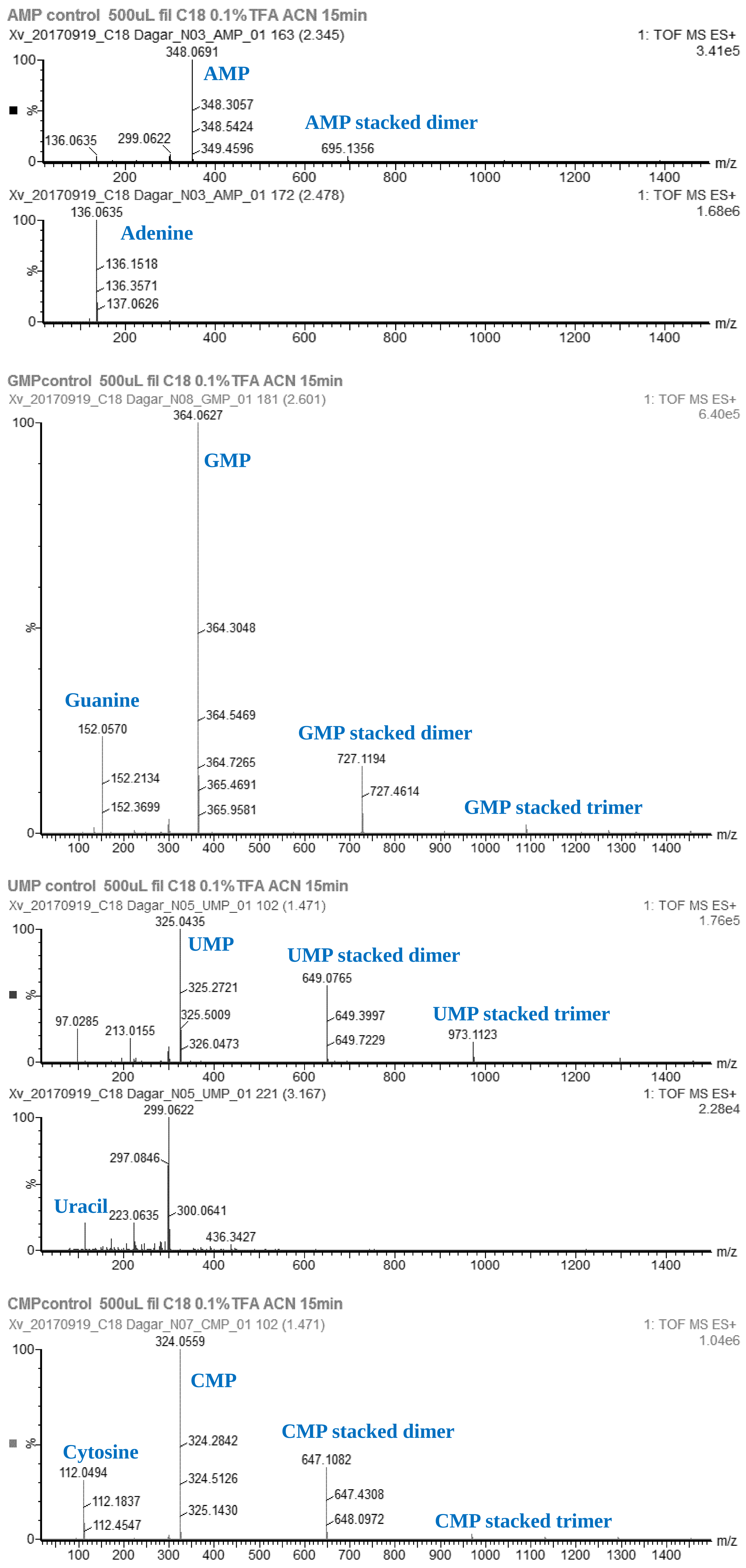

Supplementary Figure S3: Potential chemical structures of dimers and trimer based on the masses observed.

A

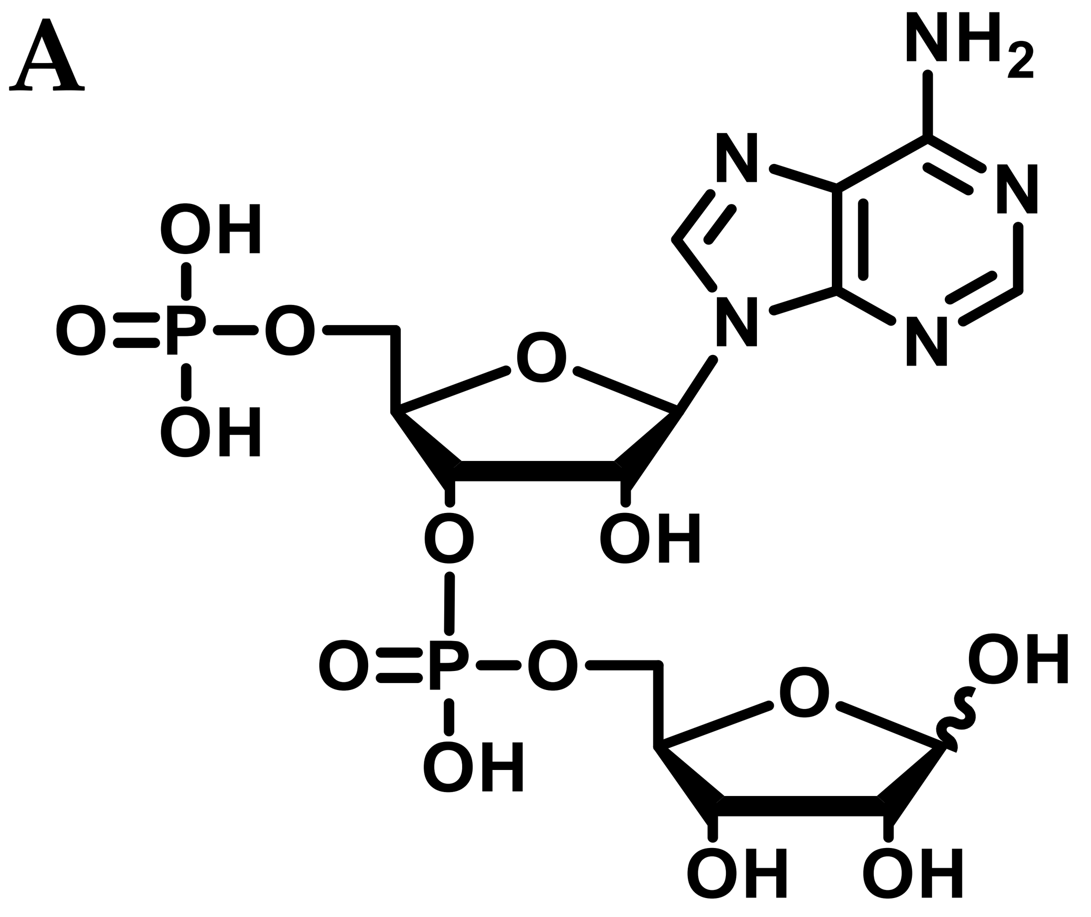

Abasic AMP Dimer

B

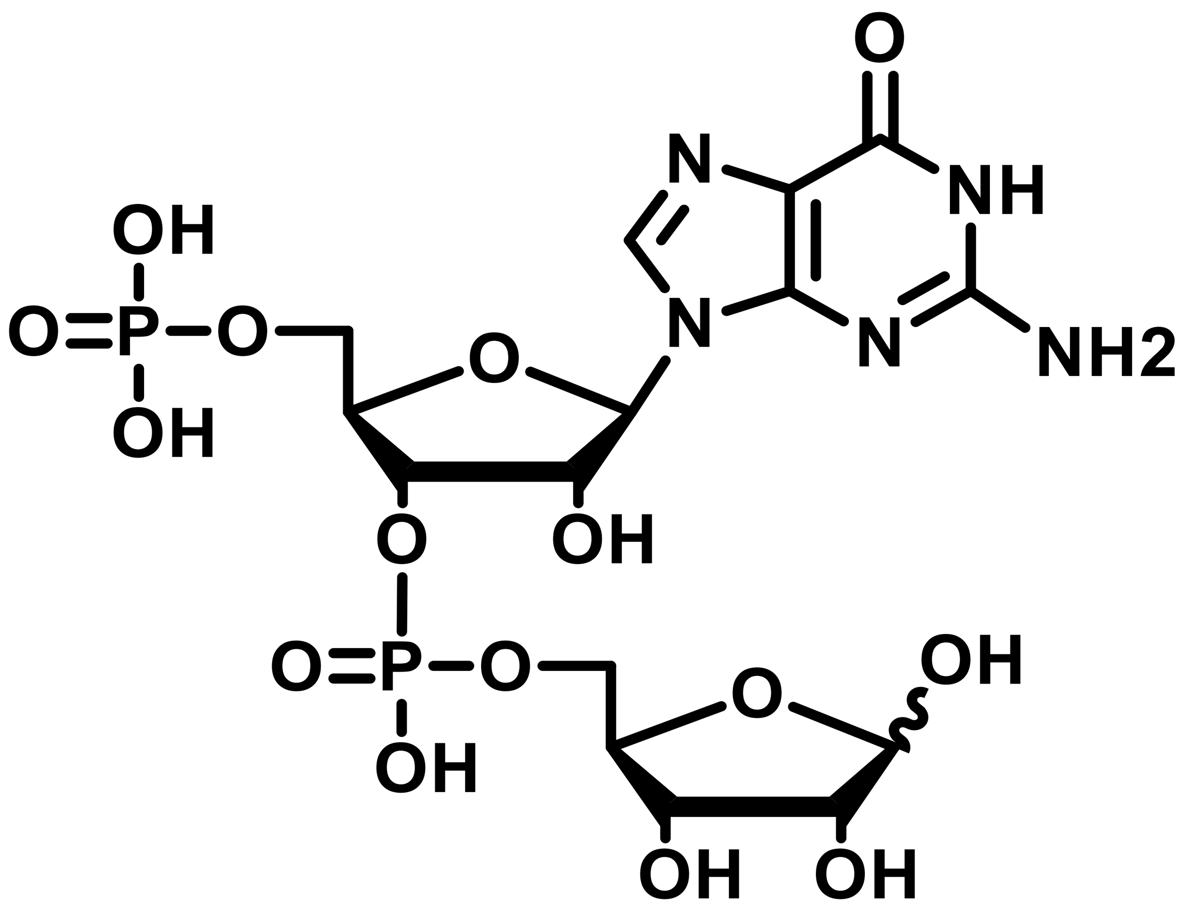

Abasic GMP Dimer

C

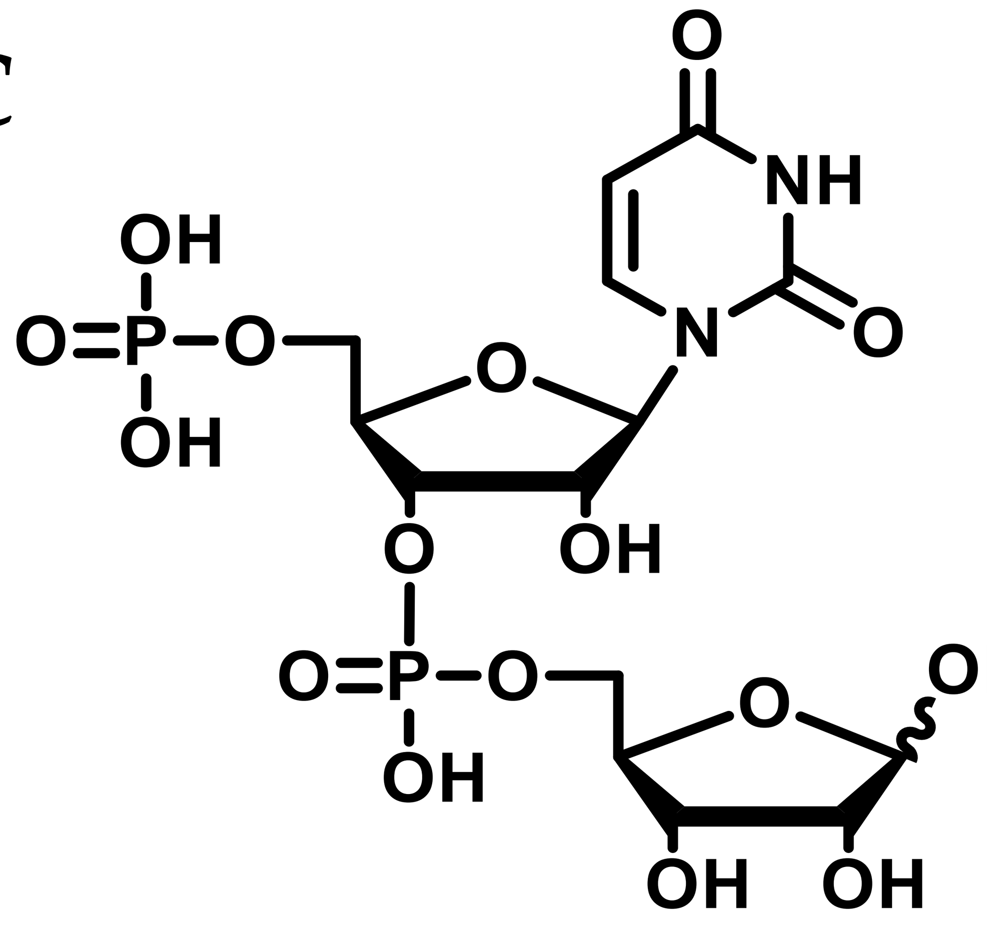

Abasic UMP Dimer

D

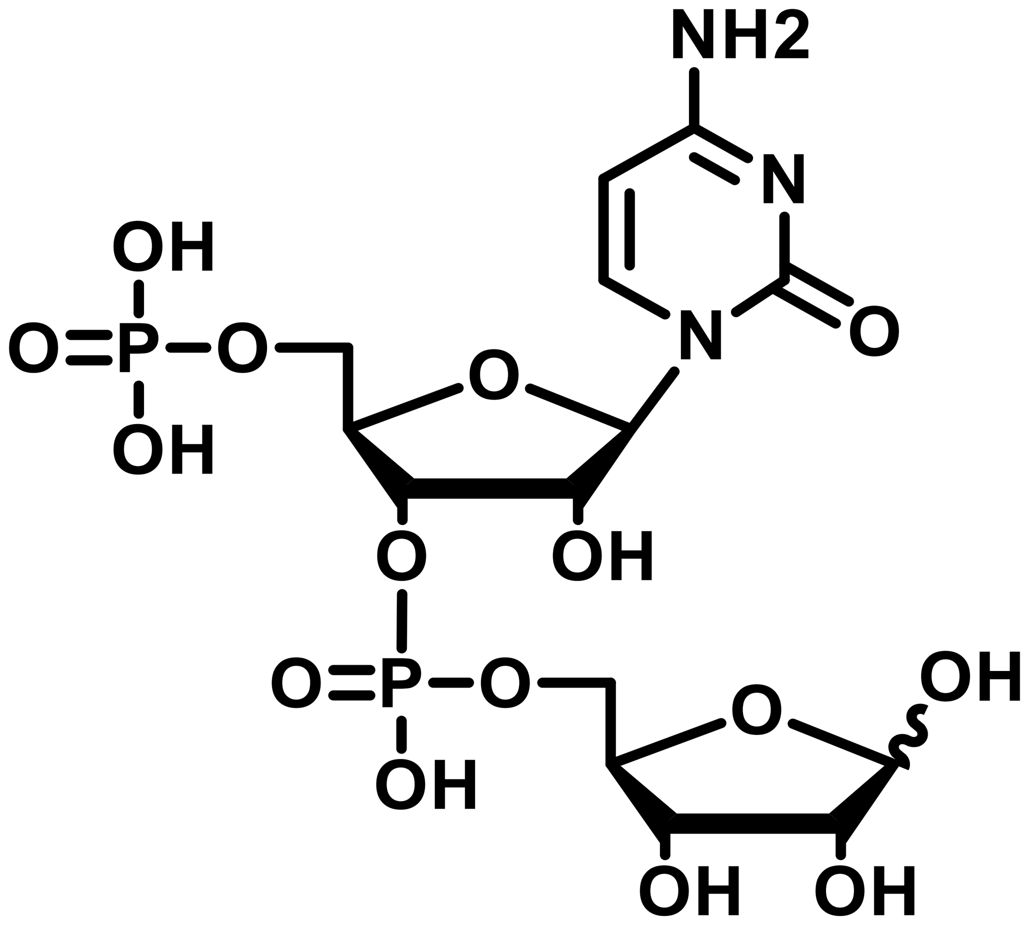

Abasic CMP Dimer

E

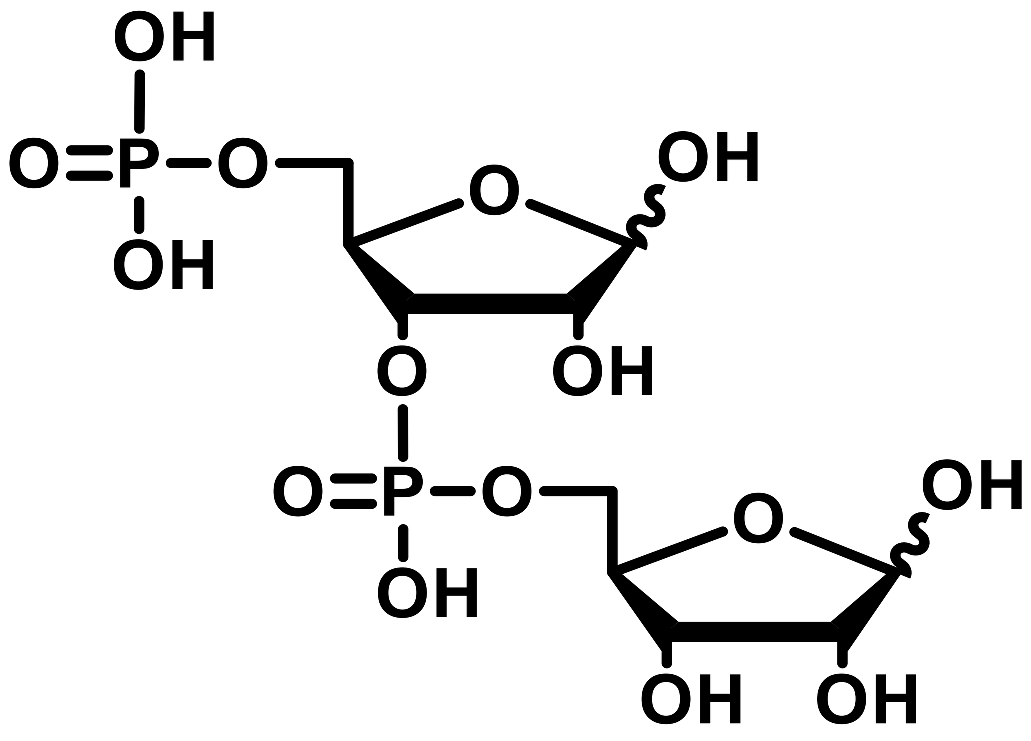

rMP Dimer

F

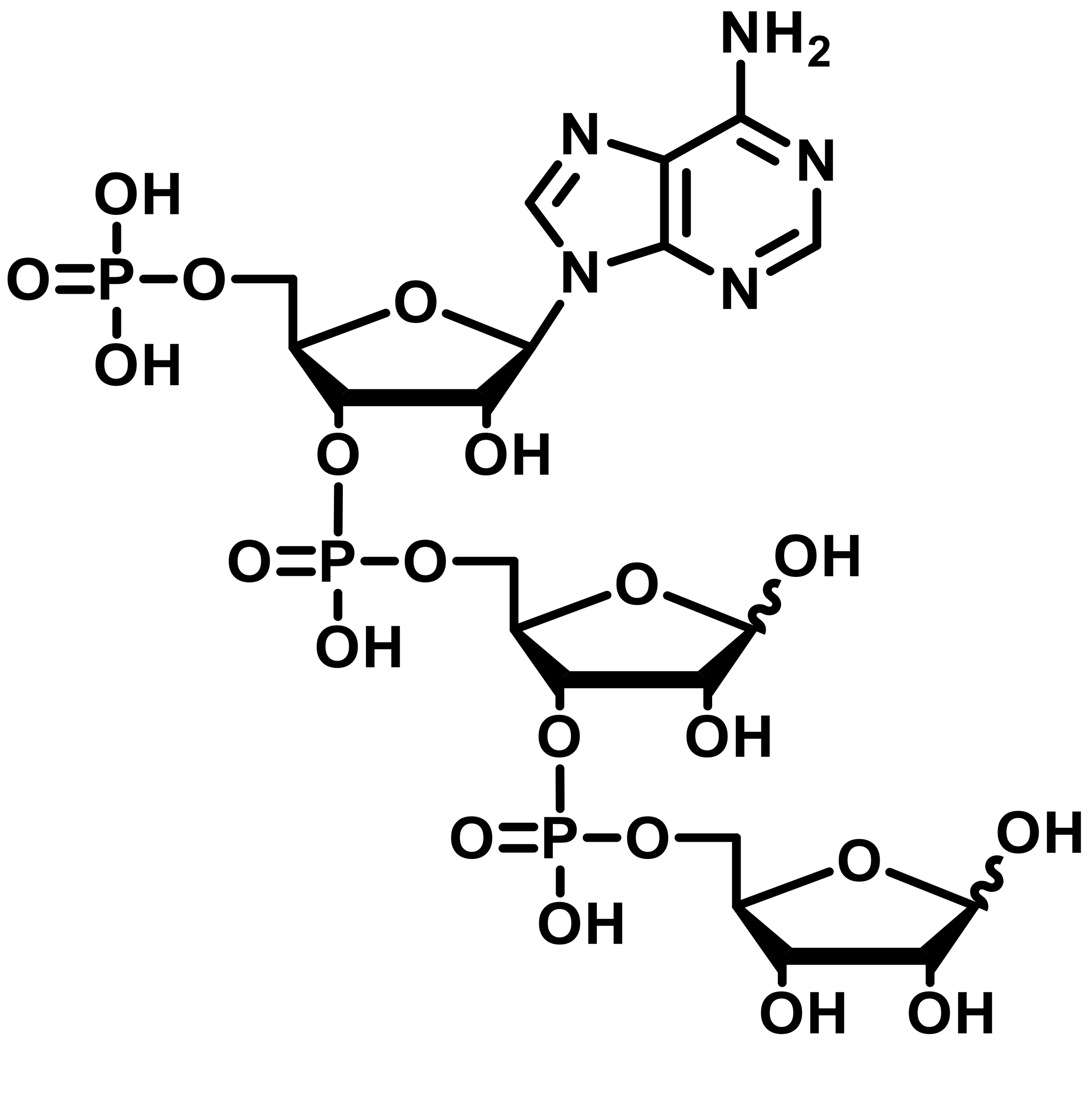

Abasic AMP Trimer

Supplementary Figure S4: Analysis of deglycosylation of nucleotides when heated at 90 °C, pH 2 under aq. solution .

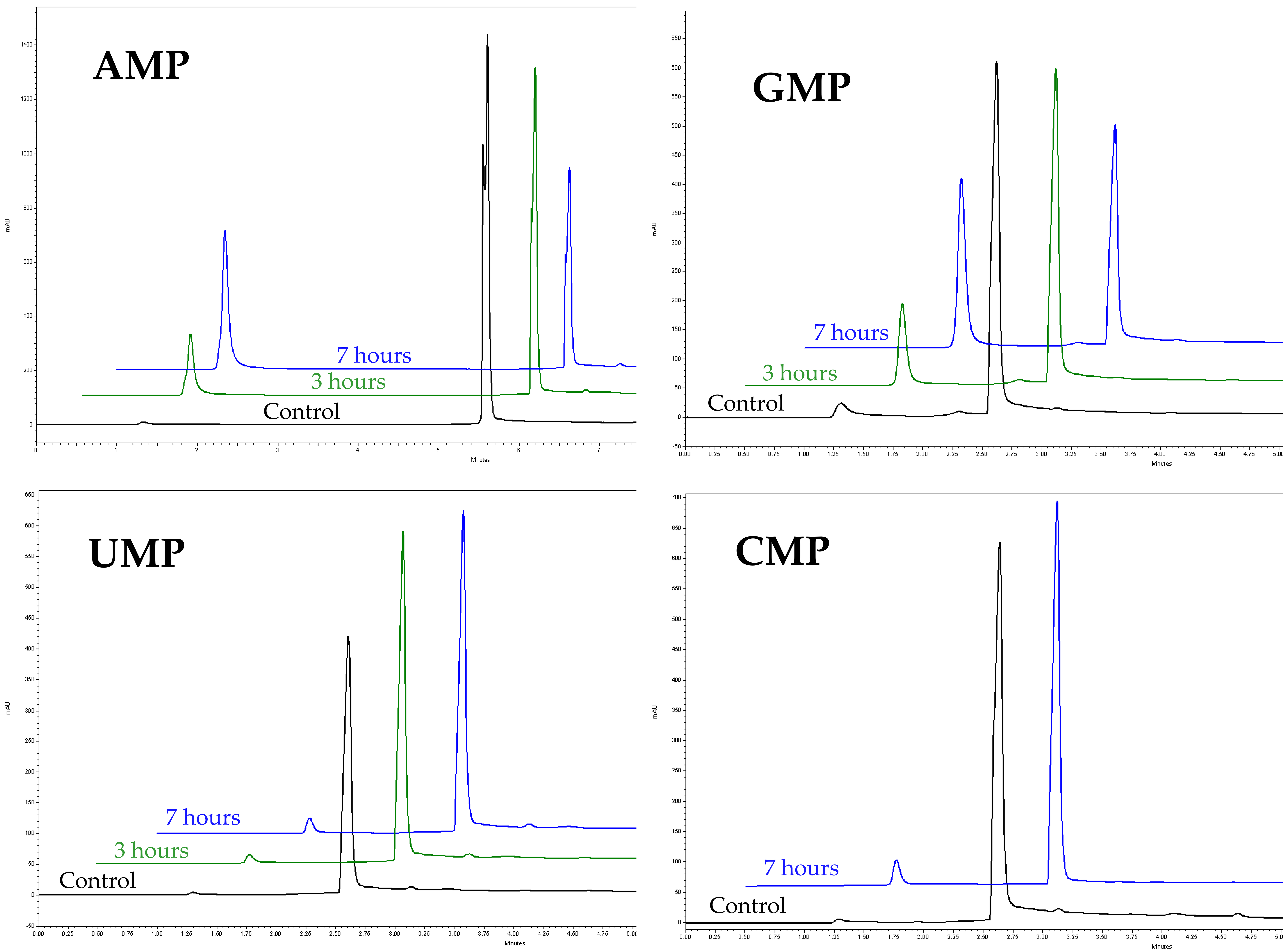

Supplement: Supplementary file 1 [file life-09-00057-s001.zip › life-528552-Supplementary Materials File 2.pdf]
